# Fexofenadine Induces ROS-Dependent Mitochondrial Dysfunction and Suppresses PI3K/AKT and MAPK Signaling in Cervical and Lung Cancer Cells

**DOI:** 10.3390/cancers18132156

**Published:** 2026-07-04

**Authors:** Ewa Trybus, Wojciech Trybus

**Affiliations:** Department of Medical Biology, Jan Kochanowski University of Kielce, Uniwersytecka 7, 25-406 Kielce, Poland

**Keywords:** fexofenadine, drug repurposing, oxidative stress, apoptosis, PI3K/AKT signaling, cervical cancer, lung cancer

## Abstract

Background/Objectives: Drug repurposing has emerged as a promising strategy for identifying novel anticancer agents among clinically established drugs. Fexofenadine, a second-generation H1 antihistamine, has been proposed as a candidate for repurposing in oncology; however, the molecular mechanisms underlying its biological activity remain insufficiently characterized. This study investigated the effects of fexofenadine on oxidative stress, mitochondrial function, apoptosis, and pro-survival signaling pathways in cervical and lung cancer cells. Methods: HeLa and A549 cancer cells, as well as non-tumorigenic Beas-2B epithelial cells, were exposed to fexofenadine under in vitro conditions. Cell viability, apoptosis, reactive oxygen species generation, mitochondrial membrane potential, DNA damage, autophagy-associated responses, and PI3K/AKT and MAPK/ERK pathway activation were assessed using flow cytometry, fluorescence microscopy, electron microscopy, and biochemical assays. Three-dimensional spheroid cultures and N-acetyl-L-cysteine rescue experiments were additionally employed to evaluate biological relevance and the contribution of oxidative stress. Results: Fexofenadine induced concentration-dependent accumulation of reactive oxygen species, mitochondrial membrane depolarization, Bcl-2 inactivation, caspase-3/7 activation, DNA damage, and apoptotic cell death in HeLa and A549 cells. Antioxidant pretreatment with N-acetyl-L-cysteine significantly reduced oxidative stress, attenuated mitochondrial dysfunction, and partially suppressed apoptosis. Fexofenadine was associated with reduced PI3K/AKT and MAPK/ERK pathway activation and promoted autophagy-associated responses. In three-dimensional spheroid cultures, treatment disrupted spheroid integrity and increased apoptotic cell death. Non-tumorigenic Beas-2B cells exhibited lower sensitivity to treatment than malignant cells. Conclusions: Fexofenadine disrupts redox homeostasis and is associated with reduced activation of pro-survival signaling pathways, resulting in oxidative stress-associated mitochondrial dysfunction and apoptosis in cancer cells. These findings provide mechanistic support for further evaluation of fexofenadine as a candidate for anticancer drug repurposing, while additional pharmacokinetic and in vivo studies are required to determine its translational relevance.

## Simple Summary

The development of new anticancer drugs is a long and costly process, which has increased interest in drug repurposing strategies. Fexofenadine is a widely used antihistamine with an established safety profile, but its potential effects on cancer cells remain insufficiently understood. In this study, we investigated the biological activity of fexofenadine in cervical and lung cancer cells and compared its effects with those observed in non-tumorigenic epithelial cells. We found that fexofenadine reduced cancer cell viability, induced programmed cell death, disrupted mitochondrial function, increased oxidative stress, and inhibited signaling pathways involved in cell survival. The drug also impaired the growth and integrity of three-dimensional tumor spheroids, which more closely resemble solid tumors than conventional cell cultures. Importantly, non-tumorigenic cells were less sensitive to treatment than cancer cells. These findings provide new insight into the biological effects of fexofenadine and support further investigation of this clinically established antihistamine as a candidate for anticancer drug repurposing.

## 1. Introduction

Lung cancer remains one of the most significant challenges in modern oncology. In 2022, it was the most frequently diagnosed cancer worldwide, with approximately 2.48 million new cases and 1.8 million deaths [1]. Cervical cancer ranks as the fourth most common cancer in women globally, with approximately 660,000 new cases and 350,000 deaths reported in the same year [2]. Despite continuous advances in prevention and therapy, both malignancies are projected to increase in incidence and mortality in the coming decades [3,4,5]. The limited long-term effectiveness of available treatments is largely attributed to tumor heterogeneity, adaptive plasticity, and the development of therapeutic resistance [6,7].

Drug resistance in cancer involves multiple mechanisms, including alterations in drug transport and metabolism, epigenetic reprogramming, enhanced DNA repair, multidrug resistance, and disruption of redox homeostasis [6,8,9,10]. Importantly, cancer cells frequently evade therapy-induced cell death by activating interconnected pro-survival signaling pathways and adaptive stress responses. Among these, the phosphatidylinositol 3-kinase/protein kinase B (PI3K/AKT) pathway plays a central role in regulating proliferation, mitochondrial stability, metabolic adaptation, and resistance to apoptosis. Aberrant activation of PI3K/AKT signaling has been documented in both non-small cell lung cancer (NSCLC) and cervical cancer and is associated with poor prognosis and reduced therapeutic response. Consequently, therapeutic strategies capable of simultaneously disrupting redox balance and pro-survival signaling networks may provide improved anticancer efficacy.

In this context, new-generation antihistamines (AHs) have attracted increasing interest. Beyond their established anti-allergic effects [11,12], histamine signaling via the H1 receptor has been implicated in tumor development, progression, and immunosuppression [13,14,15]. H1 is highly expressed in various cancer cell lines, including cervical and lung cancer models [16,17]. Elevated histamine levels and increased histidine decarboxylase (HDC) activity have been associated with tumor progression and metastatic potential [18,19], and enhanced HDC expression has been confirmed in A549 and HeLa cells [20]. Collectively, these findings suggest that antihistamines may exert anticancer effects beyond their classical anti-allergic activity, potentially involving modulation of tumor-associated signaling pathways [15,21].

Fexofenadine hydrochloride, an active metabolite of terfenadine and a highly selective H1 receptor inverse agonist, has been approved by the US Food and Drug Administration (FDA) for the treatment of allergic conditions [22]. Compared with earlier-generation antihistamines, fexofenadine demonstrates a favorable safety profile and minimal cardiovascular or central nervous system adverse effects [23,24]. While certain first-generation antihistamines have shown proapoptotic and mitochondrial-disrupting properties in experimental tumor models, their clinical applicability has been limited by safety concerns. Whether fexofenadine can modulate not only histamine-dependent signaling but also key pro-survival pathways involved in cancer progression remains insufficiently explored. Recent interest in drug repurposing has highlighted that clinically approved compounds may exert biological activities distinct from their primary therapeutic targets when evaluated in cancer models. In vitro mechanistic studies frequently employ concentration ranges exceeding clinically achieved plasma levels to identify stress-response pathways, molecular vulnerabilities, and previously unrecognized cellular targets. Although such concentrations may limit direct clinical extrapolation, these approaches can provide valuable insight into the biological actions of established drugs and support the rational design of future preclinical, pharmacokinetic, and translational investigations [25,26,27].

An important feature of malignant cells is their altered redox homeostasis and increased dependence on stress-adaptive signaling pathways that support survival under unfavorable conditions. Tumor cells frequently exhibit dysregulated PI3K/AKT and MAPK/ERK signaling, enhanced resistance to apoptosis, and increased reliance on anti-apoptotic proteins, including members of the Bcl-2 family [8,9,10,25,26,27]. These characteristics may render cancer cells particularly susceptible to agents that perturb redox balance and mitochondrial integrity. Therefore, comparative evaluation of biological responses in both tumorigenic and non-tumorigenic cellular models is important for assessing the selectivity and mechanistic relevance of potential therapeutic compounds.

The present study was designed to characterize the effects of fexofenadine on redox homeostasis, mitochondrial function, and survival signaling pathways in cervical and lung cancer cells and to compare these responses with those observed in non-tumorigenic epithelial cells. Given the functional interplay between reactive oxygen species (ROS), mitochondrial homeostasis, PI3K/AKT and MAPK/ERK signaling, and autophagy regulation, we hypothesized that fexofenadine induces a coordinated cellular stress response involving oxidative imbalance, mitochondrial dysfunction, attenuation of PI3K/AKT and MAPK/ERK signaling, and activation of apoptosis-associated pathways. Accordingly, we evaluated the impact of fexofenadine on cell viability, apoptosis, redox status, mitochondrial integrity, DNA damage, autophagy, and PI3K/AKT-MAPK signaling in HeLa and A549 cells, including three-dimensional spheroid models.

## 2. Materials and Methods

### 2.1. In Vitro Culture Conditions

HeLa and A549 cells were maintained in Dulbecco’s Modified Eagle Medium (DMEM) and Ham’s F-12 medium, respectively (Gibco, New York, NY, USA). Both media were supplemented with 10% fetal bovine serum (Biowest, Nuaillé, France) and an antibiotic–antimycotic mixture containing amphotericin B, penicillin G, and streptomycin (Corning, Manassas, VA, USA). Cultures were grown at 37 °C in a humidified incubator supplied with 5% CO_2_ (Direct Heat CO_2_ Incubator, Thermo Fisher Scientific, Waltham, MA, USA). BEAS-2B human bronchial epithelial cells were propagated in LHC-9 medium under the same environmental conditions. Cells were routinely passaged using 0.05% trypsin–EDTA, while enzymatic dissociation of BEAS-2B cultures was terminated with Defined Trypsin Inhibitor (Gibco, New York, NY, USA). For experimental treatments, cells were exposed to fexofenadine hydrochloride (C_32_H_39_NO_4_·HCl; HPLC purity > 98%; Sigma-Aldrich, St. Louis, MO, USA) at final concentrations of 150, 200, 300, 350, and 400 μM.

### 2.2. Cell Viability Assessment—MTT Assay, FDA/PI Staining

Cell viability was initially determined using the MTT reduction assay, which evaluates the metabolic activity of viable cells through the conversion of MTT into insoluble formazan crystals by mitochondrial enzymes. Following 48 h exposure to increasing concentrations of fexofenadine, cells cultured in 96-well plates (Falcon, Corning Inc., Corning, NY, USA) were incubated with MTT solution (1 mg/mL; Sigma-Aldrich, St. Louis, MO, USA) for 2 h. Subsequently, the generated formazan crystals were dissolved in dimethyl sulfoxide (DMSO; Sigma-Aldrich, St. Louis, MO, USA), and absorbance was measured at 570 nm using a Synergy 2 microplate reader (BioTek, Winooski, VT, USA). Cell viability was additionally evaluated by fluorescein diacetate/propidium iodide (FDA/PI) double staining. After 48 h treatment with 400 μM fexofenadine, cells were harvested by trypsinization and collected by centrifugation. The resulting cell pellets were incubated with fluorescein diacetate and propidium iodide (both 1 mg/mL; Sigma-Aldrich, St. Louis, MO, USA). Stained cells were mounted on microscope slides and examined using a Nikon Eclipse 80i fluorescence microscope (Nikon Instruments, Inc., Melville, NY, USA). Live cells were identified by green fluorescence resulting from intracellular conversion of FDA to fluorescein, whereas non-viable cells exhibited red fluorescence due to PI uptake through compromised plasma membranes and subsequent DNA binding. Quantitative analysis was performed by counting stained cells in 30 randomly selected microscopic fields. Mean values were calculated from three independent experiments.

### 2.3. Detection of Apoptosis

Apoptotic cell death was quantified using the Annexin V Dead Cell Kit (Merck KGaA, Darmstadt, Germany). Following 48 h exposure to fexofenadine, cells were detached with 0.25% trypsin–EDTA (Corning, Manassas, VA, USA), collected by centrifugation, and processed according to the manufacturer’s protocol. Briefly, cell suspensions were incubated with Annexin V-PE and 7-AAD staining solution for 20 min at room temperature in the absence of light. The percentage of viable, early apoptotic, late apoptotic, and necrotic cells was subsequently determined using a Muse Cell Analyzer (Merck-Millipore, Burlington, MA, USA). All experiments were carried out in three independent biological replicates.

### 2.4. Caspase-3/7 Activity Assay

Caspase-3/7 activation was evaluated using the Muse Caspase-3/7 Assay Kit (Merck-Millipore, Guyancourt, France). Following 48 h treatment with fexofenadine, cells were harvested by trypsinization and collected by centrifugation. The resulting cell pellets were incubated with 5 μL of Caspase-3/7 working reagent for 30 min at 37 °C in accordance with the manufacturer’s instructions. The proportion of cells exhibiting activated caspase-3/7 was subsequently quantified using a Muse Cell Analyzer (Merck-Millipore, Guyancourt, France). All measurements were performed in three independent experiments.

### 2.5. Assessment of Bcl-2 Protein Phosphorylation

Changes in Bcl-2 activation status were analyzed using the Muse Bcl-2 Activation Dual Detection Kit (Merck-Millipore, Guyancourt, France). The assay simultaneously detects total Bcl-2 protein and its phosphorylated form using two fluorescently labeled antibodies: anti-phospho-Bcl-2 (Ser70)-Alexa Fluor^®^ 555 and anti-Bcl-2-PE-Cy5. The relative proportion of phosphorylated Bcl-2 was determined with respect to total cellular Bcl-2 expression, allowing assessment of Bcl-2 pathway activity following fexofenadine treatment. Fluorescence signals were quantified using a Muse Cell Analyzer (Merck-Millipore, Guyancourt, France). All measurements were performed in three independent experiments.

### 2.6. Dual PI3K/MAPK Pathway Activation Assay

Activation of the PI3K/AKT and MAPK/ERK signaling pathways was evaluated using the Muse^®^ Dual PI3K/MAPK Pathway Activation Kit (Merck-Millipore, Guyancourt, France). This assay enables simultaneous detection of phosphorylated AKT (Ser473) and phosphorylated ERK1/2 (Thr202/Tyr204, Thr185/Tyr187) using fluorochrome-conjugated phospho-specific antibodies. Following 48 h exposure to 200 μM fexofenadine, cells were fixed and permeabilized according to the manufacturer’s protocol. Briefly, cell suspensions were incubated in fixation solution and subsequently treated with permeabilization buffer on ice for 10 min. Samples were then stained with 10 μL of antibody mixture and incubated for 30 min at room temperature in the dark. After washing, fluorescence signals corresponding to phosphorylated AKT and ERK1/2 were quantified using a Muse Cell Analyzer (Merck-Millipore, Burlington, MA, USA). The results were expressed as the percentage of cells exhibiting activation of the respective signaling pathways. All experiments were performed in triplicate.

### 2.7. DNA Damage Assessment

DNA damage was evaluated using the Muse^®^ Multi-Color DNA Damage Kit (Merck-Millipore, Guyancourt, France), which enables simultaneous detection of phosphorylated ATM (Ser1981) and phosphorylated histone H2A.X (Ser139), two established markers of the DNA damage response. Following treatment with fexofenadine, cells were harvested, fixed, and permeabilized according to the manufacturer’s instructions. Samples were subsequently incubated with fluorochrome-conjugated antibodies specific for phospho-ATM and phospho-H2A.X and analyzed using a Muse Cell Analyzer (Merck-Millipore, Guyancourt, France). Based on the fluorescence profiles, cells were classified as DNA damage-negative, ATM-positive, H2A.X-positive, or double-positive for both phospho-ATM and phospho-H2A.X, indicating activation of the double-strand DNA break response. All analyses were performed in three independent experiments.

### 2.8. Measurement of Reactive Oxygen Species

Intracellular oxidative stress was quantified using the Muse^®^ Oxidative Stress Kit (Merck-Millipore, Guyancourt, France). Following 48 h treatment with fexofenadine, cells were incubated with 190 μL of Muse Oxidative Stress Reagent working solution for 30 min at 37 °C according to the manufacturer’s instructions. The reagent enables discrimination between ROS-negative and ROS-positive cell populations based on oxidative conversion of the fluorescent probe. Fluorescence intensity was subsequently analyzed using a Muse Cell Analyzer (Merck-Millipore, Guyancourt, France), and the percentage of cells exhibiting elevated reactive oxygen species levels was determined. All experiments were conducted in three independent biological replicates.

### 2.9. Mitochondrial Membrane Potential Measurement

Changes in mitochondrial membrane potential (Δψm) were determined using the Muse^®^ MitoPotential Assay Kit (Merck-Millipore, Guyancourt, France). Following treatment with fexofenadine, cells were collected and incubated with MitoPotential working solution for 20 min at 37 °C. Subsequently, 5 μL of 7-aminoactinomycin D (7-AAD) was added and samples were incubated for an additional 5 min at room temperature. Fluorescence measurements were performed using a Muse Cell Analyzer (Merck-Millipore, Guyancourt, France). Based on the staining profile, the proportions of viable cells with intact mitochondrial membrane potential and cells exhibiting mitochondrial depolarization were determined. All experiments were carried out in triplicate.

### 2.10. ROS Scavenging Assay (N-acetyl-L-cysteine Rescue Experiment)

To evaluate the functional contribution of oxidative stress to fexofenadine-induced cytotoxicity, cells were pretreated with the antioxidant N-acetyl-L-cysteine (NAC) at a concentration of 5 mM (Sigma-Aldrich, St. Louis, MO, USA) for 1 h prior to fexofenadine exposure. Following pretreatment, cells were incubated with fexofenadine at a concentration of 200 µM for 48 h. The NAC-containing medium was maintained throughout the incubation period. Intracellular ROS levels were measured using the Muse^®^ Oxidative Stress Kit according to the manufacturer’s protocol. Apoptosis was assessed using the Annexin V-PE/7-AAD assay, and mitochondrial membrane potential (ΔΨm) was evaluated using the Muse^®^ MitoPotential Kit. Flow cytometric analysis was performed using the Muse^®^ Cell Analyzer. Control groups included untreated cells and cells treated with NAC alone. The experiment was performed in triplicate.

### 2.11. Assessment of Ultrastructural Changes

For transmission electron microscopy analysis, cells were initially fixed in 3% glutaraldehyde prepared in 0.1 M cacodylate buffer (pH 7.3) (Serva Electrophoresis GmbH, Heidelberg, Germany), followed by post-fixation in 2% osmium tetroxide (SPI Supplies, West Chester, PA, USA). Samples were subsequently dehydrated through a graded ethanol series (10–99.8%) and embedded in Epon 812 resin (Serva Electrophoresis GmbH, Heidelberg, Germany). Resin polymerization was carried out at 40 °C and 60 °C. Ultrathin sections were prepared using a Leica EM UC7 ultramicrotome (Leica Biosystems, Wetzlar, Germany), contrasted with uranyl acetate and lead citrate, and examined with a Tecnai G2 Spirit transmission electron microscope (FEI, Hillsboro, OR, USA) equipped with a Morada digital camera (Olympus Soft Imaging Solutions, Münster, Germany). Images were acquired and analyzed using TEM Imaging & Analysis 3.2 SP6 software (FEI Company, Hillsboro, OR, USA).

### 2.12. LC3-II Protein Level Analysis

Autophagy-associated responses were evaluated using the Muse^®^ LC3 Antibody Detection Kit (Merck-Millipore, Guyancourt, France). The assay is based on the detection of LC3-II, the lipidated form of LC3 that becomes associated with autophagosomal membranes during autophagy. Because LC3-II accumulation reflects autophagosome formation and turnover, its intracellular level is commonly used as an indicator of autophagic activity. Cells cultured in 96-well plates were exposed to fexofenadine for 48 h and subsequently incubated for 4 h with Autophagy Reagent A prepared in Earle’s Balanced Salt Solution (EBSS). This selective permeabilization step removes cytosolic LC3-I while preserving autophagosome-associated LC3-II. Following incubation, cells were washed with Hank’s Balanced Salt Solution (HBSS), harvested by trypsinization, and collected by centrifugation. Cell pellets were then stained with anti-LC3 Alexa Fluor^®^ 555 antibody in the presence of Autophagy Reagent B and incubated on ice in the dark for 30 min. Fluorescence intensity corresponding to LC3-II expression was quantified by flow cytometry. Cells maintained in serum-free medium for 4 h served as a positive control for autophagy induction. All experiments were performed in triplicate.

### 2.13. Assessment of Morphological Changes

For morphological evaluation, cells were grown on sterile glass coverslips placed in culture dishes (Falcon). Following treatment, cultures were fixed with methanol and stained using Harris hematoxylin and eosin (H&E) (Sigma-Aldrich, St. Louis, MO, USA).

After staining, samples were dehydrated through a graded ethanol series and prepared for microscopic examination. Morphological alterations were assessed using a Nikon Eclipse 80i light microscope equipped with NIS-Elements D 3.10 software (Nikon Instruments, Melville, NY, USA). A total of 3000 cells were evaluated per experimental condition in each independent experiment, yielding 9000 analyzed cells per concentration across three biological replicates.

### 2.14. Cell Cycle Analysis

After a 48 h incubation with fexofenadine, cells were fixed in ice-cold 70% ethanol and then a cell cycle assay was used (Merck-Millipore, Guyancourt, France). After staining with a reagent containing a nuclear DNA intercalating dye, propidium iodide (PI), and RNase A, cells were analyzed using a Muse analyzer (Merck-Millipore, Guyancourt, France) to determine the percentage of cells in specific cycle phases.

### 2.15. Cell Migration Assay

The extent of fexofenadine’s effect on inhibiting cell migration was assessed using a standard wound healing/scratch assay. Cells were plated and incubated for 24 h in conventional medium until a continuous confluent layer was formed. A vertical scratch (wound) was then made in this layer using a sterile pipette tip with a volume of 10 µL. The cell plates were washed twice with PBS to remove detached and damaged cells, and then incubated with fexofenadine (the control group did not contain the test compound). Microscopic images of the scratch healing at each time point (24 and 48 h) were taken with a Nikon Eclipse Ti automated microscope (Nikon Instruments Inc.) in an inverted configuration equipped with phase contrast and a cell culture system (Okolab, Pozzuoli, Italy) ensuring the maintenance of appropriate environmental parameters during observation. In dishes with control and fexofenadine-treated cells, the wound closure/migration area was assessed by measuring the distance between wound edges using a Nikon NiS Elements digital image analysis system (Nikon Instruments Inc.). Results are presented as mean values.

### 2.16. Colony Formation Assay

The clonogenic capacity of HeLa and A549 cells, measured by their proliferation and colony formation potential, was assessed. For this purpose, cells were seeded onto culture plates (100–600 cells/plate) and cultured for 24 h. Cells were then treated with various concentrations of fexofenadine (150–400 µM) and incubated at 37 °C in a 5% CO_2_ atmosphere. After 14 days, the medium was removed, and the resulting cell colonies were fixed in 3.7% paraformaldehyde for 30 min and stained with 0.05% crystal violet. Colonies were counted (>30 cells) and imaged. Results are presented as mean values.

### 2.17. 3D Spheroid Model Study

Three-dimensional (3D) cell cultures, such as spheroids, were designed as an alternative to traditional 2D cultures because they provide a more physiological model that mimics and replicates tissue architecture in vivo. They are used to evaluate the potential effects of anticancer drugs, enabling the assessment of drug penetration, efficacy, and toxicity under more realistic conditions [28]. 3D spheroids allow researchers to measure changes in spheroid size and cell viability, and observe effects such as apoptosis and necrosis [29]. After 4 days of growth, spheroids (formed by the hanging drop technique) were measured in diameter using a Nikon NiS Elements digital image analysis system (Nikon Instruments Inc.). Spheroids larger than 500 μm in diameter were used for this study because they are believed to mimic the pathophysiological conditions found in solid tumors, including a central hypoxic area and a proliferative gradient [30,31]. Live and dead cells within spheroids were visualized using fluorescein diacetate (FDA) and propidium iodide (PI) staining [27,28]. Briefly, spheroids were incubated with the fluorescent dyes (Sigma-Aldrich, St. Louis, MO, USA) for 10 min at 37 °C and immediately examined using a Nikon Ti inverted fluorescence microscope (Nikon Instruments, USA). Appropriate filter sets were applied for detection of FDA (excitation 480 nm, emission 520 nm) and PI (excitation 535 nm, emission > 610 nm) fluorescence. The procedure was repeated in three independent experiments. Quantitative assessment of apoptosis in spheroid cultures was subsequently performed using the Annexin V Dead Cell Kit (Merck KGaA, Darmstadt, Germany) following the protocol described above for monolayer cultures.

### 2.18. Statistical Analysis

All experiments were performed in at least three independent biological replicates. Quantitative data are presented as mean ± standard error (SE). Statistical significance was evaluated using one-way analysis of variance (ANOVA) followed by Tukey’s post hoc multiple-comparison test to correct for multiple testing. Differences were considered statistically significant at *p* < 0.05. Statistical analyses were performed using Statistica 13.3 software (StatSoft, Kraków, Poland).

## 3. Results

### 3.1. Effect of Fexofenadine on the Beas-2B Cell Line

In contrast to cancer cell lines, non-tumorigenic Beas-2B cells exhibited markedly lower susceptibility to fexofenadine-induced apoptosis (Supplementary Figure S1). At 200 µM, the percentage of Annexin V–positive cells reached 18.5%, compared with 49.6% in HeLa and 37.07% in A549 cells. Similarly, MTT analysis demonstrated preserved metabolic activity in Beas-2B cells, with viability exceeding 80% at 200 µM. Even at 400 µM, apoptotic levels and metabolic suppression remained significantly lower than in malignant cell models. These findings indicate that non-tumorigenic Beas-2B cells were substantially less sensitive to fexofenadine treatment than HeLa and A549 cells, supporting a degree of preferential cytotoxicity toward malignant cells under the experimental conditions used.

### 3.2. Fexofenadine Reduces the Viability of HeLa and A549 Cells

**Figure 1:**
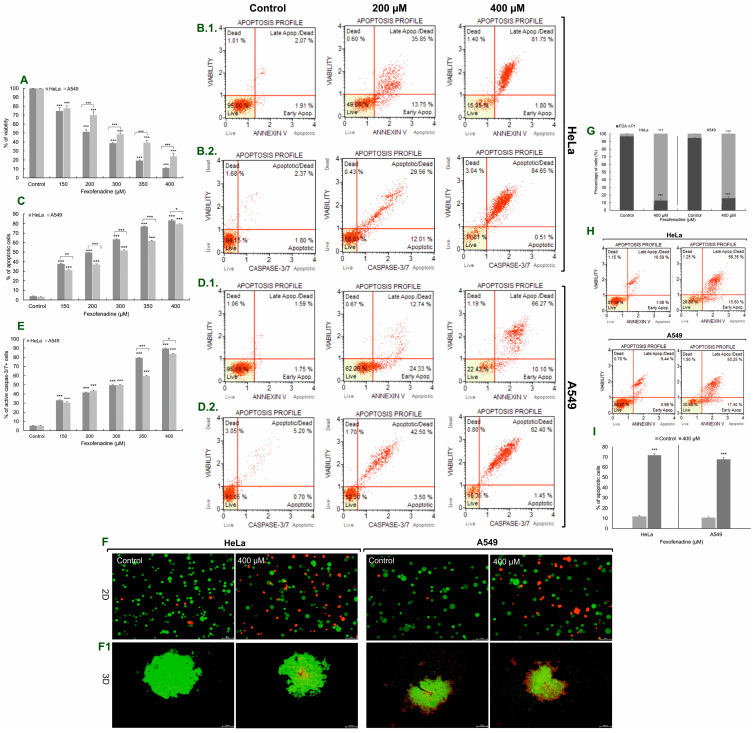
Proapoptotic effect of fexofenadine. HeLa and A549 cell lines treated for 48 h with fexofenadine at concentrations of 150 µM, 200 µM, 300 µM, 350 µM and 400 µM. Cell viability was assessed by the MTT reduction assay (**A**). The level of apoptosis was determined by the Annexin V-PE/7-AAD assay. Live cells (Annexin V-PE-/7-AAD-), early-apoptotic (Annexin V-PE+/7-AAD-), late-apoptotic (Annexin V-PE+/7-AAD+), dead (Annexin V-PE-/7-AAD+). The activity level of caspases 3/-7 was assessed using the Caspase-3/7 Kit assay. Live (caspase 3/7-/7-AAD-), early-apoptotic (caspase 3/7+/7-AAD-), late-apoptotic (caspase 3/7+/7-AAD+), dead (caspase 3/7-/7-AAD+) cells. Representative histograms illustrating the distribution of apoptotic (**B1**,**D1**) and caspase-positive (**B2**,**D2**) cells for the HeLa and A549 lines. Fexofenadine concentration-dependent percentage distribution of apoptotic (**C**) and caspase-positive (**E**) cells. Representative fluorescence microscopy images of HeLa and A549 cells treated with 400 µM fexofenadine and stained with fluorescein diacetate/propidium iodide (FDA/PI) ((**F**); magnification 200×), and spheroids (3D model) stained with FDA/PI ((**F1**); magnification 40×). Green fluorescence indicates FDA-positive (live) cells, while red fluorescence indicates PI-positive (dead) cells. Areas showing overlapping green and red fluorescence appear yellow/orange, indicating colocalization of FDA and PI signals. Percentage of FDA-positive and PI-positive cells determined by fluorescence staining in the 2D model (**G**). Representative Annexin V-PE/7-AAD flow cytometry plots of spheroids (**H**) and the corresponding percentage distribution of apoptotic cell populations (**I**). Data are presented as mean ± SE from three independent biological experiments (*n* = 3). Statistical significance was assessed using one-way ANOVA followed by Tukey’s multiple-comparison test. * *p* < 0.05, ** *p* < 0.01, *** *p* < 0.001.

The MTT assay showed a statistically significant (*p* ≤ 0.0001) and progressive decrease in the ability of cells to reduce MTT dye with increasing concentration of the test compound (Figure 1A). Compared to the control (which was assumed to be 100%), already at the lowest concentration (150 µM), the viability of HeLa and A549 cells decreased to approximately 75% and 78%, respectively. At subsequent concentrations of 200 µM and 300 µM, a further reduction in viability was observed, amounting to 51.4% and 38.69% for HeLa and 69.98% and 48.69% for A549, respectively. The intensity of changes was confirmed at concentrations of 350 µM and 400 µM, with the lowest viability result recorded at 400 µM, i.e., 11% for HeLa and 24% for A549. The observed reduction in MTT reduction capacity indicates a concentration-dependent decrease in cellular metabolic activity and viability following fexofenadine treatment. The cytotoxic effect of fexofenadine on both tested cell lines was also confirmed by microscopic analysis of cells stained with fluorescent FDA/PI (Figure 1F,G). It was shown that the tested compound at a concentration of 400 µM induced an increase in the percentage of dead cells (above 80%; *p* ≤ 0.0001) in HeLa and A549 cell lines, which exhibited a red fluorescence color resulting from propidium iodide labeling. In contrast, control (live) cells were characterized by green fluorescence emission resulting from fluorescein diacetate. These observations were consistent with the MTT assay results and confirmed the pronounced cytotoxic effect of fexofenadine in both cancer cell lines. Based on MTT viability data, the estimated IC_50_ values were approximately 211 µM for HeLa cells and 293 µM for A549 cells, whereas the corresponding value for non-tumorigenic Beas-2B cells was approximately 391 µM. These findings further support the lower susceptibility of non-tumorigenic epithelial cells to fexofenadine-induced cytotoxicity. Representative flow-cytometric histograms corresponding to intermediate concentrations are provided in Supplementary Figure S2.

### 3.3. Fexofenadine Induces Apoptosis in HeLa and A549 Cells

Exposure to fexofenadine resulted in a significant increase in the number of apoptotic cells. At a concentration of 150 µM, apoptotic cells in the HeLa line constituted 37.72% (*p* ≤ 0.0001), increasing to approximately 50% at 200 µM (*p* ≤ 0.0001) (Figure 1(B1),C). The highest percentage of apoptotic cells was observed at concentrations of 350 µM and 400 µM, reaching 77.09% and 83.55%, respectively (*p* ≤ 0.0001), with a predominance of late-apoptotic cells. Similarly, treatment of A549 cells with fexofenadine significantly increased apoptosis to 31.24% and 37.07% at 150 µM and 200 µM, respectively (*p* ≤ 0.0001) (Figure 1(D1),C). Further increases in concentration enhanced the apoptotic response, reaching 51.8% at 300 µM, 61.91% at 350 µM, and approximately 80% at 400 µM (*p* ≤ 0.0001). To further characterize the mechanism of cell death, caspase-3/7 activity was evaluated. Fexofenadine induced a concentration-dependent increase in the proportion of cells with activated caspase-3/7 in both cell lines, with the highest values observed at 400 µM, exceeding 89% in HeLa cells and 84% in A549 cells (Figure 1(B2,D2),E). These findings indicate that fexofenadine-induced apoptosis is associated with activation of the executioner caspase cascade.

### 3.4. Fexofenadine Exerts Cytotoxic Effects in Spheroids (3D Model)

FDA/PI staining revealed distinct viability patterns in control and treated spheroids (Figure 1(F1)). In control spheroids, two characteristic layers were observed: an outer layer composed predominantly of viable cells (green FDA fluorescence) and a central region containing dead cells (red PI fluorescence), reflecting the typical architecture of multicellular spheroids. Following 48 h exposure to 400 µM fexofenadine, spheroids exhibited extensive structural disruption, increased PI-positive staining, and the appearance of numerous small cell aggregates, indicating loss of spheroid integrity. Flow cytometric Annexin V analysis confirmed the pronounced proapoptotic effect of fexofenadine in the three-dimensional model (Figure 1H,I). The percentage of apoptotic cells increased to 71.9% in HeLa spheroids and 67.7% in A549 spheroids, with a predominance of late-apoptotic cells, whereas control spheroids exhibited only 11.65% and 10.45% apoptotic cells, respectively. These findings demonstrate that fexofenadine retains substantial cytotoxic and proapoptotic activity in three-dimensional tumor spheroids, supporting its biological activity in a model that more closely resembles tumor architecture than conventional monolayer cultures.

### 3.5. Fexofenadine Increases Bcl-2 Phosphorylation Associated with Loss of Anti-Apoptotic Activity

**Figure 2:**
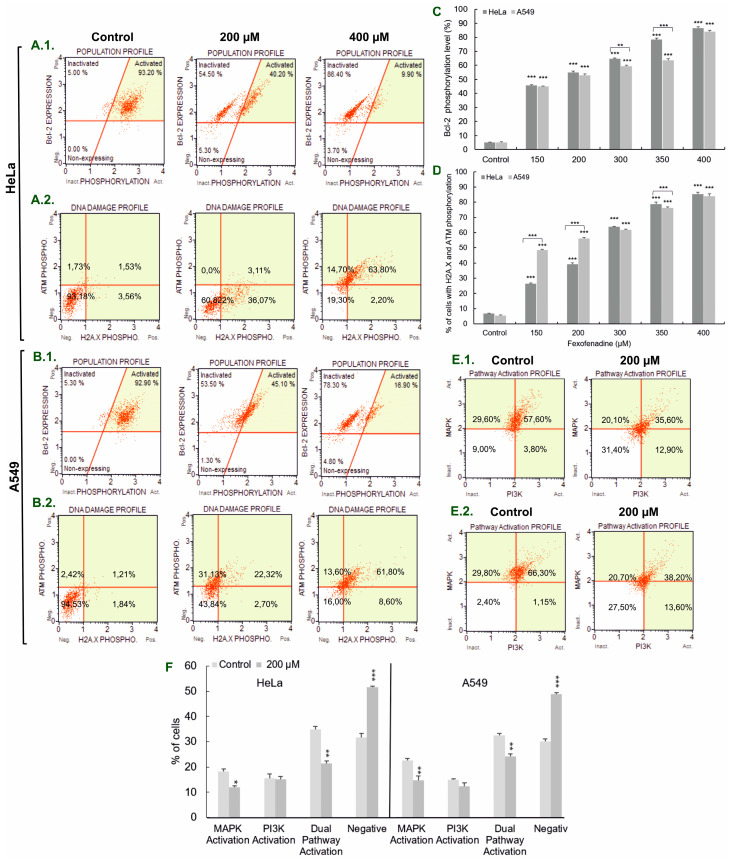
Bcl-2 protein inactivation, DNA damage, and modulation of PI3K/AKT and MAPK/ERK signaling pathways in HeLa and A549 cells following fexofenadine treatment. Representative histograms showing the distribution of cells with Bcl-2 protein inactivation (**A1**,**B1**) and DNA damage (**A2**,**B2**) after 48 h exposure to fexofenadine (150–400 µM). Quantitative analysis of cells with inactivated Bcl-2 protein (**C**) and cells exhibiting double-stranded DNA breaks, determined by dual activation of ATM and H2A.X (**D**). Representative flow cytometry plots illustrating the effect of fexofenadine (200 µM, 48 h) on PI3K/AKT and MAPK/ERK signaling pathways in HeLa (**E1**) and A549 (**E2**) cells, based on the detection of phosphorylated AKT (p-AKT) and phosphorylated ERK1/2 (p-ERK). (**F**) Quantitative analysis of signaling pathway activation expressed as the percentage distribution of cell populations positive for p-AKT and/or p-ERK. Signaling pathway analysis was performed at 200 µM, corresponding to the approximate IC_50_ concentration in HeLa cells and a submaximal cytotoxic concentration in A549 cells. Data are presented as mean ± SE from three independent biological experiments (*n* = 3). Statistical significance was assessed using one-way ANOVA followed by Tukey’s multiple-comparison test; * *p* < 0.05, ** *p* < 0.01, *** *p* < 0.001.

The proapoptotic activity of fexofenadine was further supported by analysis of Bcl-2 phosphorylation status. A concentration-dependent increase in the proportion of cells exhibiting Bcl-2 phosphorylation associated with loss of anti-apoptotic activity was observed in both tested cell lines (Figure 2(A1,B1),C). In HeLa cells, the percentage of affected cells reached 45.73% and 54.9% (*p* ≤ 0.0001) following treatment with 150 µM and 200 µM fexofenadine, respectively. Comparable effects were observed in A549 cells, where the corresponding values were 45.1% and 53% (*p* ≤ 0.0001). Further increases in fexofenadine concentration resulted in a progressive enhancement of Bcl-2 inactivation. At 300 µM and 350 µM, the percentage of cells exhibiting phosphorylated Bcl-2 reached 64.83% and 78.3% (*p* ≤ 0.0001) in HeLa cells and 59.38% and 63.46% (*p* ≤ 0.0001) in A549 cells, respectively. The highest proportion of cells with phosphorylated/inactivated Bcl-2 was observed following treatment with 400 µM fexofenadine, exceeding 80% in both cell lines (*p* ≤ 0.0001). These findings indicate that fexofenadine promotes functional inactivation of the anti-apoptotic Bcl-2 pathway, which may contribute to mitochondrial destabilization and activation of the intrinsic apoptotic cascade.

### 3.6. Fexofenadine Induces ATM and H2A.X Phosphorylation Indicative of DNA Damage

Treatment with fexofenadine resulted in a concentration-dependent increase in the proportion of cells exhibiting ATM activation, H2A.X phosphorylation, and concurrent ATM/H2A.X positivity, indicative of double-strand DNA breaks (Figure 2(A2,B2),D). In HeLa cells, the percentage of cells with DNA damage increased progressively from 26.46% at 150 µM to 39.18% at 200 µM, 63.8% at 300 µM, 78.76% at 350 µM, and 85.3% at 400 µM (*p* ≤ 0.0001). A549 cells exhibited a similar but more pronounced response. At the lowest concentration tested (150 µM), cells displaying ATM/H2A.X activation already constituted 48.6% of the population. Further increases in fexofenadine concentration resulted in progressive DNA damage, reaching 56.16% at 200 µM, 61.7% at 300 µM, 76.2% at 350 µM, and 84% at 400 µM (*p* ≤ 0.0001).

These findings demonstrate that fexofenadine induces substantial DNA damage in both cancer cell lines, as evidenced by activation of the ATM-mediated DNA damage response and increased H2A.X phosphorylation, consistent with the presence of double-strand DNA breaks.

### 3.7. Modulation of the PI3K/MAPK Signaling Pathway by Fexofenadine in the Context of Apoptosis Markers

The activity of PI3K and MAPK signaling pathways was evaluated by flow cytometry using a four-quadrant analysis distinguishing non-activated cells (LL), MAPK activation (UL), PI3K activation (LR), and dual PI3K/MAPK activation (UR). The signaling profiles were analyzed in parallel with Annexin V staining, caspase 3/7 activation, and Bcl-2 expression levels. In HeLa control cells, the majority of the population exhibited simultaneous activation of PI3K and MAPK pathways (UR: 57.6%), while only a small fraction of cells was classified as non-activated (LL: 9.0%). Single-pathway activation was limited (UL: 29.6%; LR: 3.8%) (Figure 2(E1),F). This profile corresponds to the low baseline apoptotic fraction detected by Annexin V (~4%) and caspase 3/7 (~5%), as well as preserved Bcl-2 expression, indicating active pro-survival signaling under control conditions. Exposure of HeLa cells to 200 µM resulted in a marked redistribution of signaling populations. The proportion of non-activated cells increased from 9.0% to 31.4%, while the dual-activated fraction decreased from 57.6% to 35.6%. A moderate PI3K-only population (LR: 12.9%) and MAPK-only population (UL: 20.1%) remained detectable. This shift toward reduced dual pathway activation and increased non-activated cells coincided with a substantial rise in Annexin V-positive cells (49.6%), increased caspase 3/7 activation (41.7%), and reduced Bcl-2 levels (54.9%), indicating that attenuation of coordinated PI3K/MAPK signaling accompanies mitochondrial apoptotic progression in HeLa cells. In A549 control cells, dual pathway activation was even more pronounced (UR: 66.3%), with a minimal fraction of non-activated cells (LL: 2.4%) (Figure 2(E2),F). MAPK-only activation accounted for 29.8%, whereas PI3K-only activation was low (1.5%). This signaling profile is consistent with low apoptotic indices measured by Annexin V (~3%) and caspase 3/7 (~5%) and preserved Bcl-2 expression, reflecting strong constitutive pro-survival signaling.

Following treatment with 200 µM, A549 cells demonstrated a shift in pathway distribution characterized by an increase in non-activated cells to 27.5% and a reduction in dual activation to 38.2%. Notably, a PI3K-only fraction (13.6%) and MAPK-only fraction (20.7%) persisted. These changes paralleled a moderate increase in Annexin V positivity (37.1%), caspase 3/7 activation (43.7%), and decreased Bcl-2 expression (53%). Compared with HeLa cells, attenuation of dual pathway activation was less pronounced, suggesting partial maintenance of survival signaling in A549 cells under treatment conditions. Collectively, these findings indicate that fexofenadine-induced apoptosis is accompanied by a redistribution of signaling populations characterized by reduced coordinated PI3K/MAPK activation and expansion of the non-activated fraction. This effect was more pronounced in HeLa cells, which also exhibited greater susceptibility to apoptosis. Although the present study does not establish whether PI3K/AKT and MAPK/ERK attenuation represents a primary molecular target or a downstream consequence of oxidative stress, the observed association between pathway suppression, Bcl-2 inactivation, caspase activation, and apoptotic cell death supports a functional link between survival signaling attenuation and mitochondrial apoptosis.

### 3.8. Fexofenadine Induces Oxidative Stress in the Tested Cells

**Figure 3:**
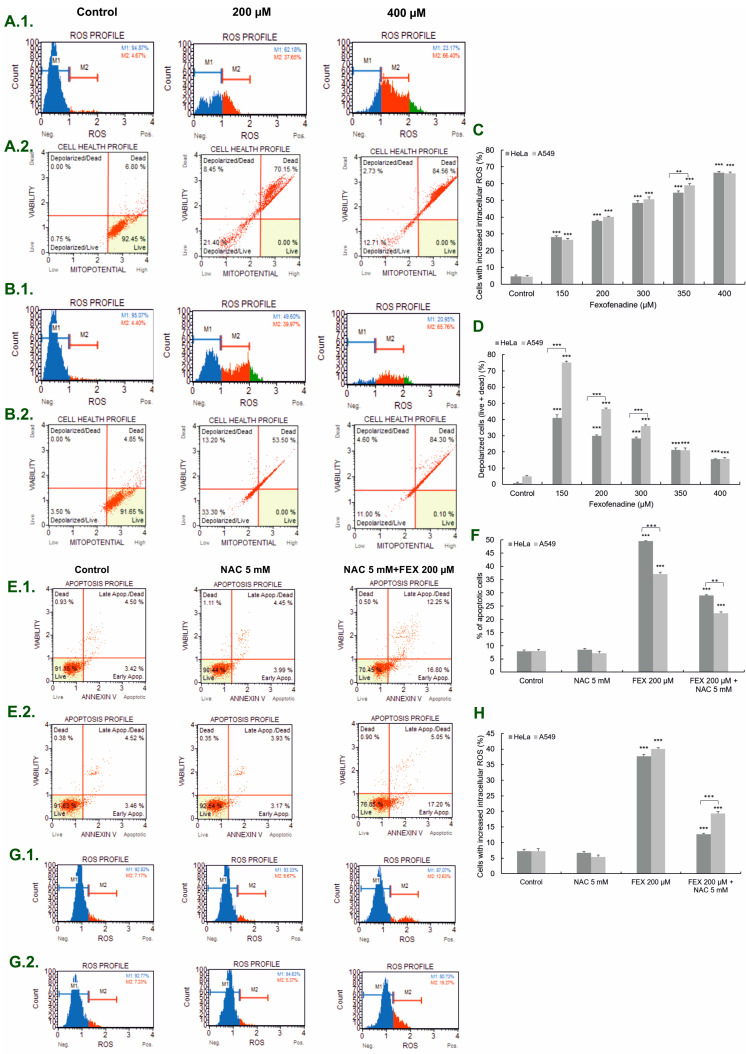
Oxidative stress–associated mitochondrial dysfunction and the effect of ROS scavenging in HeLa and A549 cells exposed to fexofenadine. Concentration-dependent changes in intracellular reactive oxygen species (ROS) levels (**A1**,**B1**) and mitochondrial membrane potential (ΔΨm) (**A2**,**B2**) in HeLa and A549 cells after 48 h exposure to fexofenadine (150–400 µM). In the histograms, cells producing ROS are designated as ROS (+), whereas unstained cells are designated as ROS (−). Cells exhibiting mitochondrial membrane depolarization are designated as ΔΨm (−). Quantitative analysis of ROS-positive cells (**C**) and cells with mitochondrial membrane depolarization (**D**). Representative flow cytometry histograms illustrating the effect of N-acetyl-L-cysteine (NAC; 5 mM, 1 h) pretreatment on apoptosis (**E1**,**E2**) and intracellular ROS generation (**G1**,**G2**) in HeLa and A549 cells exposed to fexofenadine (200 µM, 48 h). NAC was administered 1 h before fexofenadine treatment and maintained throughout the incubation period. Quantitative analysis of Annexin V-positive (**F**) and ROS-positive (**H**) cell populations in control, NAC alone, fexofenadine (200 µM), and NAC + fexofenadine groups. Data are presented as mean ± SE from three independent biological experiments (*n* = 3). Statistical significance was assessed using one-way ANOVA followed by Tukey’s multiple-comparison test; ** *p* < 0.01, *** *p* < 0.001.

Fexofenadine-induced oxidative stress in cervical and lung cancer cells was confirmed by a statistically significant increase in reactive oxygen species (ROS) levels at all tested concentrations (*p* ≤ 0.0001) (Figure 3(A1,B1),C). At concentrations of 150 µM and 200 µM, ROS-positive cells constituted 28.11% and 37.7% in HeLa cells and 26.48% and 40.07% in A549 cells, respectively. Further increases in fexofenadine concentration intensified ROS generation. In HeLa cells, ROS-positive populations reached 48.51% at 300 µM, 54.58% at 350 µM, and 66.43% at 400 µM. Similarly, in A549 cells, ROS-positive populations increased to 50.61%, 58.91%, and 66.08% at 300 µM, 350 µM, and 400 µM, respectively. The assay measured total intracellular ROS and therefore did not distinguish between mitochondrial and cytosolic ROS pools.

Elevated ROS production was accompanied by a reduction in mitochondrial membrane potential (ΔΨm) (Figure 3(A2,B2),D). In HeLa cells, the proportion of cells exhibiting mitochondrial depolarization reached 41% at 150 µM (*p* ≤ 0.0001). At 200 µM and 300 µM, approximately 30% of cells displayed reduced mitochondrial membrane potential. At higher concentrations (350–400 µM), the proportion of depolarized cells declined to 21.3% and 15.44%, respectively (*p* ≤ 0.0001). Comparable effects were observed in A549 cells, where the percentages of cells with reduced mitochondrial membrane potential were 75.05%, 46.5%, 36.1%, 21.05%, and 15.6% at 150 µM, 200 µM, 300 µM, 350 µM, and 400 µM, respectively.

The apparent decrease in the proportion of cells exhibiting mitochondrial depolarization at the highest concentrations (350–400 µM) is likely attributable to progression toward late-stage apoptosis and secondary necrosis. Under these conditions, a substantial fraction of cells may no longer retain sufficient mitochondrial integrity for reliable assessment of membrane potential. Consequently, mitochondrial depolarization should be interpreted as an early-to-intermediate apoptotic event rather than a strictly linear dose-dependent marker. Collectively, these findings demonstrate that fexofenadine induces pronounced oxidative stress accompanied by mitochondrial dysfunction, supporting a mechanistic association between ROS accumulation and activation of the intrinsic apoptotic pathway.

### 3.9. ROS Scavenging Partially Attenuates Fexofenadine-Induced Apoptosis in HeLa and A549 Cells

To determine whether oxidative stress functionally contributes to fexofenadine-induced cytotoxicity, we performed rescue experiments using the antioxidant N-acetyl-L-cysteine (NAC; 5 mM) (Figure 3(G1,G2),H). Cells were pretreated with NAC for 1 h prior to exposure to fexofenadine (200 µM) and incubated for 48h under continuous antioxidant supplementation. The concentration of 200 µM was selected based on its previously demonstrated ability to induce substantial but non-terminal cytotoxic effects. As shown earlier, fexofenadine markedly increased intracellular ROS levels and apoptotic cell populations in both cell lines. Consistent with these findings, exposure to 200 µM fexofenadine elevated the percentage of ROS-positive cells to 37.7% in HeLa and 40.07% in A549 cells, compared with 7.17% and 7.23% in respective controls. NAC alone did not significantly alter basal ROS levels (6.67% in HeLa; 5.37% in A549), confirming the absence of intrinsic pro-oxidant or cytotoxic effects under the applied conditions. Importantly, NAC pretreatment significantly reduced fexofenadine-induced ROS accumulation. In HeLa cells, ROS-positive populations decreased from 37.7% to 12.63% following combined NAC and fexofenadine treatment, whereas in A549 cells ROS levels declined from 40.07% to 19.27%. Although ROS levels in the combined treatment groups remained moderately elevated relative to control, the magnitude of oxidative stress was markedly attenuated.

A similar pattern was observed for apoptosis. Fexofenadine treatment increased Annexin V-positive cells to 49.6% in HeLa and 37.07% in A549 cells, compared with 7.92% and 7.99% in control groups. NAC alone did not induce apoptosis (8.44% in HeLa; 7.1% in A549). Notably, antioxidant pretreatment significantly reduced fexofenadine-induced apoptosis, lowering Annexin-positive populations to 29.05% in HeLa and 22.25% in A549 cells. Although apoptosis was not completely abolished, the substantial reduction observed following ROS neutralization indicates that oxidative stress plays a mechanistically relevant role in fexofenadine-induced apoptotic signaling. The incomplete rescue effect suggests that, in addition to ROS-dependent mechanisms, parallel stress-associated pathways may also contribute to cell death induction. Collectively, these findings demonstrate that redox imbalance represents an important functional mediator of fexofenadine cytotoxicity in cervical and lung cancer cells.

### 3.10. Fexofenadine Induces Ultrastructural Alterations Associated with Mitochondrial Dysfunction and Autophagy

**Figure 4:**
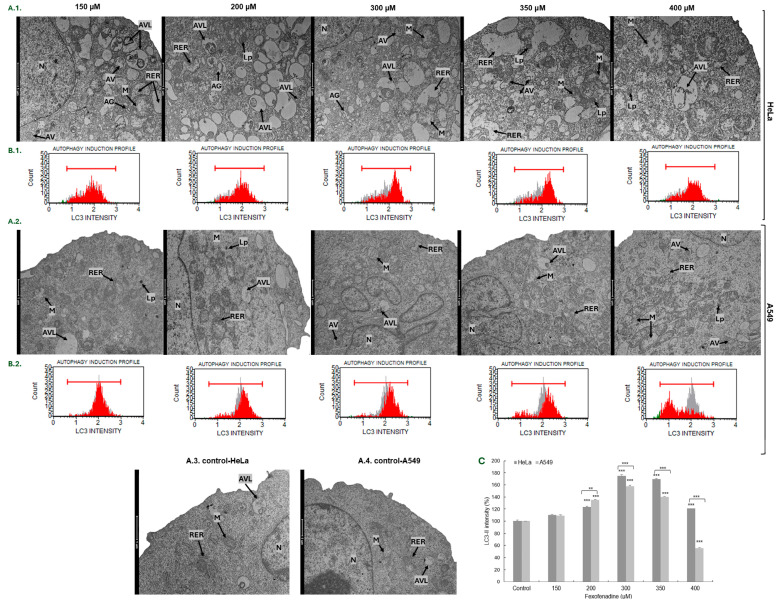
Ultrastructural alterations and modulation of LC3-II expression in HeLa and A549 cells following 48 h exposure to fexofenadine. (**A1**) Representative transmission electron microscopy images of HeLa cells treated with fexofenadine. At 150–300 µM, cells exhibited swollen mitochondria with a cleared matrix, dilated rough endoplasmic reticulum (RER) cisternae, Golgi apparatus disorganization, and numerous primary lysosomes, autophagosomes, and autophagolysosomes. At 350–400 µM, more pronounced mitochondrial damage, extensive RER dilation, and features consistent with endoplasmic reticulum stress were observed. (**A2**) Representative transmission electron microscopy images of A549 cells exposed to fexofenadine. At 150–200 µM, mitochondria with a cleared matrix, primary lysosomes, autophagosomes, and autophagolysosomes were observed. At 300–400 µM, ultrastructural alterations became more pronounced and included swollen mitochondria with disrupted cristae, dilated RER cisternae, and nuclear abnormalities, including irregular morphology and fragmentation. (**A3**,**A4**) Untreated control HeLa and A549 cells showing normal cellular ultrastructure. Abbreviations: N—nucleus; M—mitochondria; AV—autophagic vacuoles; AVL—autophagolysosomes; LP—primary lysosomes; AG—Golgi apparatus; RER—rough endoplasmic reticulum. Magnification: 16,500×. (**B1**,**B2**) Representative flow cytometry histograms showing LC3-II expression in HeLa and A549 cells after 48 h exposure to fexofenadine (150–400 µM). (**C**) Quantitative analysis of LC3-II expression presented as relative fluorescence intensity compared with untreated controls. Data are presented as mean ± SE from three independent biological experiments (*n* = 3). Statistical significance was assessed using one-way ANOVA followed by Tukey’s multiple-comparison test; ** *p* < 0.01, *** *p* < 0.001.

Transmission electron microscopy revealed pronounced ultrastructural alterations in both cancer cell lines following fexofenadine treatment (Figure 4). Compared with control HeLa cells (Figure 4(A3)), exposure to 150–300 µM fexofenadine (Figure 4(A1)) resulted in mitochondrial swelling with matrix clearing, dilation of rough endoplasmic reticulum cisternae, and dispersion of Golgi apparatus structures. In parallel, numerous primary lysosomes, double-membrane autophagosomes, and autophagolysosomes at various stages of degradation were observed, indicating activation of autophagy-associated processes.

Treatment with higher concentrations of fexofenadine (350–400 µM) further intensified these ultrastructural alterations. Mitochondria displayed marked swelling, loss of cristae organization, and extensive matrix clearing, consistent with severe mitochondrial injury. These changes were accompanied by pronounced dilation of rough endoplasmic reticulum cisternae occupying large areas of the cytoplasm, suggesting activation of cellular stress responses. At the same time, the number of autophagosomes and autophagolysosomes appeared reduced compared with lower concentrations, coinciding with the predominance of apoptotic features.

Similar ultrastructural changes were observed in A549 cells. Relative to control cells (Figure 4(A4)), treatment with 150–200 µM fexofenadine (Figure 4(A2)) induced mitochondrial swelling and accumulation of lysosomal and autophagic structures. At concentrations of 300–400 µM, the alterations became more pronounced and involved extensive rough endoplasmic reticulum dilation, nuclear deformation and fragmentation, as well as severe mitochondrial damage characterized by swelling, matrix clearing, and disruption of mitochondrial cristae.

Collectively, the ultrastructural findings support the occurrence of mitochondrial dysfunction, cellular stress responses, autophagy-associated processes, and apoptotic progression following fexofenadine exposure.

### 3.11. Fexofenadine Modulates LC3-II Levels and Autophagy-Associated Responses

The ultrastructural observations were complemented by analysis of LC3-II levels, a marker associated with autophagosome formation (Figure 4(B1,B2),C). In both HeLa and A549 cells, treatment with fexofenadine resulted in concentration-dependent alterations in LC3-II fluorescence intensity. Increased fluorescence intensity (red histograms) relative to untreated controls (gray histograms) indicated accumulation of LC3-II-positive structures following drug exposure. No statistically significant changes in LC3-II levels were observed at 150 µM in either cell line. However, treatment with 200 µM and 300 µM fexofenadine significantly increased LC3-II fluorescence intensity to 123.29% and 175.08% of control values in HeLa cells and to 135.48% and 157.66% in A549 cells, respectively (*p* ≤ 0.0001). At higher concentrations (350–400 µM), LC3-II levels progressively declined. In HeLa cells, fluorescence intensity decreased to 169.7% and 121.1% of control values, whereas in A549 cells it decreased to 140.2% and 55.64%, respectively (*p* ≤ 0.0001). The biphasic pattern of LC3-II modulation suggests that lower and intermediate concentrations of fexofenadine promote autophagy-associated responses, whereas higher concentrations shift the cellular response toward extensive cytotoxicity and apoptotic cell death. These findings are consistent with the ultrastructural observations and support the involvement of autophagy-related processes in the cellular response to fexofenadine treatment.

### 3.12. Fexofenadine Increases Vacuolization and Apoptotic Changes—Analysis at the Light Microscopy Level (H&E Staining)

**Figure 5:**
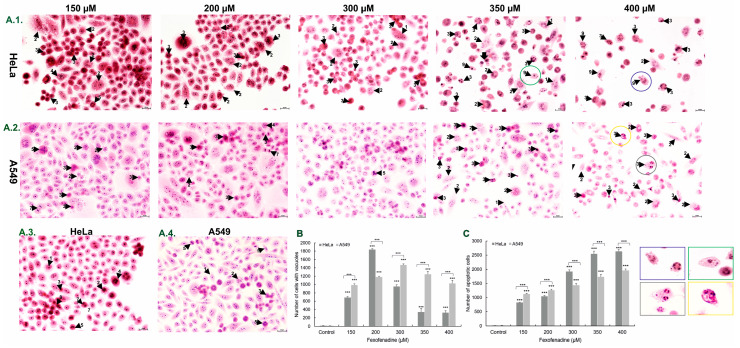
Vacuolization and apoptotic changes induced by 48 h fexofenadine exposure. Representative images of H&E-stained cells. Magnification: 400×. HeLa (**A1**) and A549 (**A2**) cells exposed to fexofenadine (150–400 µM) exhibited concentration-dependent cytoplasmic vacuolization (visible as unstained, sharply demarcated cytoplasmic areas) and apoptotic morphology characterized by cell shrinkage, eosinophilic cytoplasm, chromatin condensation, and nuclear fragmentation. Untreated control cells (**A3**,**A4**) displayed normal morphology and mitotic activity. Symbols: 1—interphase; 2—vacuolated cell; 3—apoptotic cell; 4—prophase; 5—metaphase; 6—anaphase; 7—telophase; 8—cytokinesis; 9—cell exhibiting both vacuolization and apoptotic features. Cells simultaneously displaying extensive vacuolization and apoptotic morphology are highlighted and shown at higher magnification. Quantitative analysis of vacuolated (**B**) and apoptotic (**C**) cells following fexofenadine treatment. Data are presented as mean ± SE from three independent biological experiments (*n* = 3). Statistical significance was assessed using one-way ANOVA followed by Tukey’s multiple-comparison test; *** *p* < 0.001.

Compared to control cells (Figure 5(A3,A4)), analysis of HeLa and A549 cell lines exposed to 48 h of fexofenadine (Figure 5(A1,A2),B,C) showed concentration-dependent vacuolization changes (vacuoles were visible as unstained spaces clearly demarcated from the cytoplasm) and changes typical of apoptosis (cells were characterized by chromatin condensation and fragmentation, strong shrinkage and darkly stained cytoplasm). Already at the lowest concentration of the compound of 150 µM, an increase in the number of vacuolated cells to 692 (*p* ≤ 0.0001) and apoptotic cells to 826 (*p* ≤ 0.0001) was observed in HeLa cells. For the A549 line, higher results were obtained at the same concentration: 987 vacuolated cells (*p* ≤ 0.0001) and 1128 apoptotic cells (*p* ≤ 0.0001). Fexofenadine at a concentration of 200 µM clearly enhanced both vacuolated and apoptotic changes in both cell lines tested. In the HeLa line, vacuolated cells constituted almost 61% (1839 cells; *p* ≤ 0.0001), which was the highest result observed, while the number of apoptotic cells was 1046 (*p* ≤ 0.0001), representing approximately 35% of all cells analyzed. In contrast, the A549 line responded with a smaller increase in the number of vacuolated cells to 1179 (*p* ≤ 0.0001), while apoptotic changes were more pronounced (1257 cells; *p* ≤ 0.0001). At the 300 µM concentration, a different trend was observed when comparing the two cell lines. Morphological analysis of the HeLa line cells revealed a sharp decrease in the number of vacuolated cells, which constituted 32% of all analyzed cells (953 cells; *p* ≤ 0.0001), with a simultaneous progressive increase in the number of apoptotic cells to 1925 (64%) (*p* ≤ 0.0001). In the case of the A549 line, however, an increase in both vacuolation and apoptosis was observed. Vacuolated and apoptotic cells remained in a 1:1 ratio, as confirmed by an increase in their number to 1471.6 (*p* ≤ 0.0001) and 1440.5 (*p* ≤ 0.0001), respectively. The action of high concentrations of fexofenadine 350 µM and 400 µM on cervical cancer cells resulted in a significant reduction in the number of vacuolated cells to 339 (*p* ≤ 0.0001) and 326 (*p* ≤ 0.0001), respectively, in favor of the number of apoptotic cells, amounting to 2544 (*p* ≤ 0.0001) and 2628 (*p* ≤ 0.0001), respectively, which constituted over 80% of the pool of all analyzed cells. In lung cancer cells, there was a slight reduction in the number of vacuolated cells to 1246 (*p* ≤ 0.0001) (350 µM) and to 1023.5 (*p* ≤ 0.0001) (400 µM). Simultaneously, a gradual increase in the number of apoptotic cells was observed, reaching 1731 (57.7%) (*p* ≤ 0.0001) at a concentration of 350 µM and 1959 (65.3%) (*p* ≤ 0.0001) at a concentration of 400 µM, which was the highest result obtained for this line. Characteristically, in the case of both tested lines, at concentrations of 350–400 µM, the presence of cells with simultaneous features was observed, such as increased vacuolization of the cytoplasm and a pyknotic cell nucleus with partial chromatin condensation or a nucleus with features of disintegration (shown in panels A1 and A2, also in magnification). Collectively, these findings support a concentration-dependent transition from stress-associated vacuolization to apoptotic cell death, which is consistent with the results obtained using Annexin V, caspase-3/7, LC3-II, and transmission electron microscopy analyses.

### 3.13. Fexofenadine Inhibits the Migration of HeLa and A549 Cells

**Figure 6:**
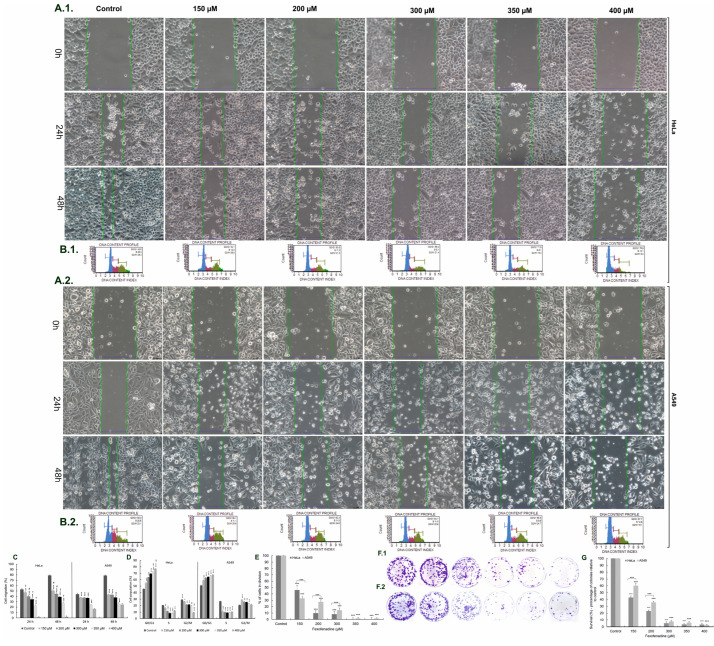
Antiproliferative effect of fexofenadine. Microscopic images showing the change in scratch width at different time intervals as a result of cell migration in the HeLa (**A1**) and A549 (**A2**) cell groups loaded with fexofenadine at concentrations of 150–400 µM. Untreated cell monolayers with the same vertical scratches served as negative controls. Green lines define the cell-free area (wound margins), whereas the blue horizontal arrows indicate the measured wound width used for quantitative analysis of cell migration. Magnification × 200. Representative histograms from flow cytometry analysis showing the distribution of cells in the cell cycle of HeLa (**B1**) and A549 (**B2**) cells exposed to fexofenadine at concentrations of 150–400 µM for 48 h. (**C**) Quantification of the effect of fexofenadine on the migratory potential of HeLa and A549 cells. Results represent mean values from three independent experiments ± SE. Statistical differences were confirmed at ** *p* ≤ 0.01; *** *p* ≤ 0.001. The symbol # denotes a statistically significant change (*p* ≤ 0.001) in wound size in the group of HeLa cells exposed to 200 μM fexofenadine compared to A549 cells under influence on the same concentration. The symbol † denotes a statistically significant change (*p* ≤ 0.01) in wound size in the group of HeLa cells treated with 150 μM and 300–400 μM fexofenadine compared to A549 cells loaded with the same concentration of the test compound. (**D**) Percentage distribution of HeLa and A549 cells in different phases of the cell cycle, indicating cell blocking in the G0/G1 phase, * *p* ≤ 0.05. (**E**) Changes in mitotic index indicating a decrease in cell division capacity by fexofenadine. Results represent means from three independent experiments ± SE. Statistical differences were confirmed at *** *p* ≤ 0.001. (**F1**,**F2**) Representative clonogenic assays illustrating the long-term effects of fexofenadine on colony formation by HeLa and A549 cells after 14 days of culture. (**G**) Quantitative analysis of survival fractions. Data are presented as mean ± SE from three independent biological experiments (*n* = 3). Statistical differences were confirmed at *** *p* ≤ 0.001.

The “torn wound” creates a cell-free space through which the rest of the cultured cells can migrate, mimicking the wound-healing process. Fexofenadine-induced changes in the migratory capacity of HeLa and A549 cells were observed at two time points—after 24 and 48 h (Figure 6(A1,A2),C). In the control group of HeLa cells (where the width of the “torn wound” at time 0 was set at 280.23 µm), a 53% reduction (approx. 132 µm) in wound width was observed after 24 h, and virtually complete healing of the monolayer (approx. 79%) occurred after 48 h (58.88 µm). Fexofenadine treatment, on the other hand, clearly delayed wound healing. The width of the “torn wound” at time 0 was set at 270.96 µm. The wound closure rate for the 150 µM concentration after 24 and 48 h was 47.48% (142.3 µm) (*p* ≤ 0.01) and 50.7% (137.39 µm) (*p* ≤ 0.0001), respectively. Under the influence of concentrations of 200 µM and 300 µM, the percentage of cell migration was determined to be 40.08% (163.01 µm) (*p* ≤ 0.0001) and 34.44% (179.92 µm) (*p* ≤ 0.0001) after 24 h of exposure to the compound and 44.88% (149.93 µm) (*p* ≤ 0.0001) and 39.01% (167.38 µm) (*p* ≤ 0.0001) after 48 h of exposure. In turn, under the influence of higher concentrations of fexofenadine, cervical cancer cells showed a significant delay in their ability to migrate into the empty space, for 350 µM the reduction in wound width was 22.85% (215.35 µm) (*p* ≤ 0.0001) after 24 h of exposure and 27.54% (202.27 µm) (*p* ≤ 0.0001) after 48 h of exposure, while for 400 µM it was only 1.13% (276.42 µm) (*p* ≤ 0.0001) after 24 h and 2.31% (273.14 µm) (*p* ≤ 0.0001) after 48 h.

Untreated A549 cells migrated and reduced the torn wound width (which at time point 0 was 275 µm) in the range of 44% (154 µm) to 78.78% (58.34 µm) after 24 and 48 h of fexofenadine exposure, respectively. However, cell migration was reduced at concentrations of 150 µM–300 µM. The lowest concentration of 150 µM reduced the torn wound width by 37.71% (170.35 µm/24 h) (*p* ≤ 0.0001) and 43.29% (155.38 µm/48 h) (*p* ≤ 0.0001). As a result of the action of fexofenadine at concentrations of 200 µM and 300 µM, the percentage of cell migration at two time points was determined to be 37.66% (173.92 µm/24 h) and 43.13% (158.65 µm/48 h) and 36.95% (173.37 µm/24 h) and 37.94% (170.65 µm), respectively. However, high concentrations of the compound 350 µM and 400 µM resulted in a slight reduction in the width of the “torn wound”, which, depending on the exposure time, was 24.29% (210.45 µm) and 16.62% (228.44 µm) in the case of 24 h exposure and 26.26% (204.99 µm) and 24.78% (206.08 µm) in the case of 48 h exposure, respectively. Collectively, these findings demonstrate that fexofenadine reduces the migratory capacity of both HeLa and A549 cells in a concentration-dependent manner.

### 3.14. Fexofenadine Blocks Cells in the G0/G1 Phase and Reduces the Mitotic Index

Flow cytometric analysis demonstrated a concentration-dependent accumulation of cells in the G0/G1 phase accompanied by a reduction in the S-phase population, indicating induction of G0/G1 cell-cycle arrest following fexofenadine treatment (Figure 6(B1,B2),D). For the HeLa and A549 lines, at the lowest concentration of the tested compound of 150 µM, 54.7% (*p* ≤ 0.0001) and 59.1% (*p* ≤ 0.0001) of cells were arrested in this phase, respectively. In both tested cell lines, subsequent concentrations of 200 µM and 300 µM resulted in statistically significant results (*p* ≤ 0.0001), which were found in the range of approximately 63% to 69.2% (with the highest result for HeLa cells). The highest increase in the cell population in the G0/G1 phase was observed for cervical cancer cells at concentrations of 350 µM and 400 µM, which was 71.81% (*p* ≤ 0.0001) and 76.91% (*p* ≤ 0.0001), respectively. In the case of lung cancer cells, these values were lower, i.e., 65.9% (*p* ≤ 0.0001) for 350 µM and 67.5% (*p* ≤ 0.0001) for 400 µM fexofenadine.

Furthermore, exposure of HeLa and A549 cells to fexofenadine at concentrations of 150–400 µM caused a significant reduction in mitotic activity. The changes observed were statistically significant (*p* ≤ 0.0001) at all concentrations analyzed relative to the control set at 100% (Figure 6E). HeLa cells were more sensitive to the tested compound, as already at a concentration of 150 µM their ability to divide decreased significantly to 46%, and as a result of exposure to subsequent concentrations, progressive changes in the mitotic index were observed, which for concentrations of 200–300 µM was in the range of 8–10%, while for 350–400 µM it was only 0.6–0.3%, which was the lowest result recorded in this experiment. In the case of A549 cells, for concentrations of 150 µM and 200 µM, the mitotic index was determined to be 33.18% and 27.58%, respectively, and these were the highest values recorded, because under the influence of subsequent concentrations, the division capacity of these cells decreased significantly, reaching a maximum of 15% for 300 µM and 2% for 350–400 µM. Collectively, the results suggest that fexofenadine impairs cancer cell proliferation through induction of G0/G1 cell-cycle arrest and a marked reduction in mitotic activity.

### 3.15. Fexofenadine Reduces the Clonogenic Potential of Hela and A549 Cells

Clonogenic assays demonstrated a concentration-dependent reduction in colony-forming capacity following fexofenadine treatment (Figure 6(F1,F2),G). In HeLa cells, exposure to 150 µM and 200 µM fexofenadine reduced colony formation to 43% and 23% of control values, respectively (*p* ≤ 0.0001). In A549 cells, the corresponding values were approximately 23% and 36% of control levels (*p* ≤ 0.0001). Further increases in fexofenadine concentration (300–400 µM) resulted in a profound suppression of clonogenic growth, reducing colony formation to 3–5% of control values in HeLa cells and to approximately 3–8% in A549 cells (*p* ≤ 0.0001). These findings indicate that fexofenadine markedly impairs the long-term proliferative and colony-forming capacity of both cancer cell lines.

### 3.16. Preincubation of Cells with Chloroquine Enhances the Cytotoxicity of Fexofenadine

**Figure 7:**
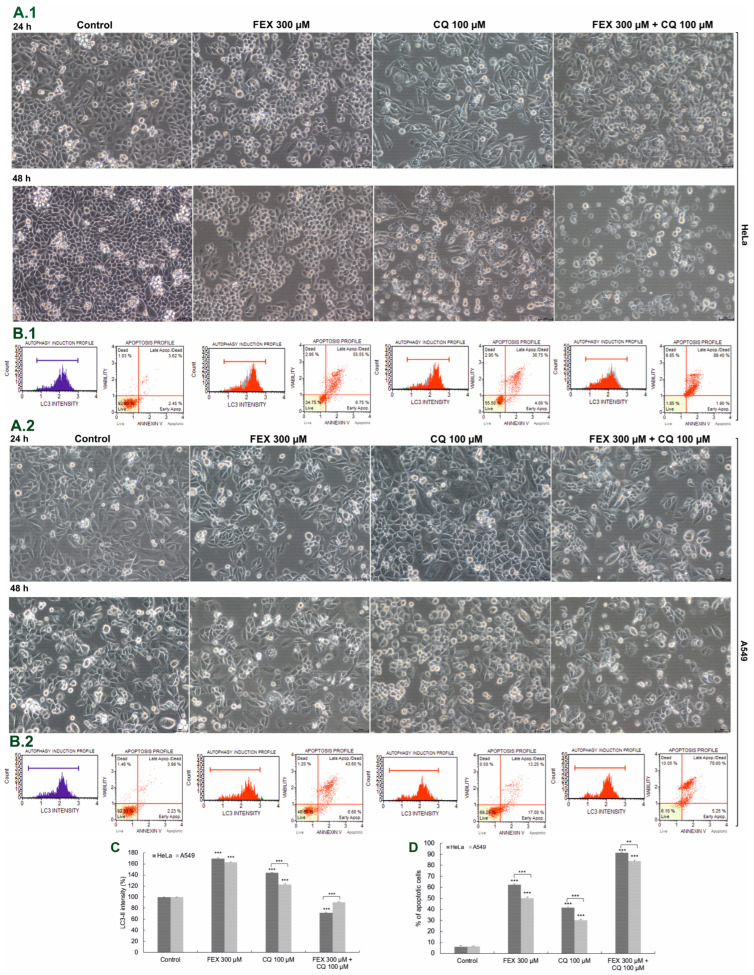
Effect of combined treatment with fexofenadine and chloroquine on apoptosis induction in HeLa and A549 cells. Cells were treated with fexofenadine (FEX, 300 μM), chloroquine (CQ, 100 μM), or a combination of both compounds (FEX 300 μM + CQ 100 μM) for 24 and 48 h. (**A1**,**A2**) Representative phase-contrast images of HeLa and A549 cells (magnification 200×). Cytoplasmic vacuolization was observed following treatment with chloroquine and fexofenadine. Combined exposure increased the number of vacuolated cells after 24 h, consistent with chloroquine-mediated modulation of autophagy-associated processes. After 48 h, combined treatment enhanced apoptotic morphology, characterized by an increased number of rounded and shrunken cells accompanied by a reduction in vacuolated cells. (**B1**,**B2**) Representative flow cytometry plots showing LC3-II expression and Annexin V-PE/7-AAD staining in HeLa and A549 cells after 48 h exposure. (**C**) Quantitative analysis of LC3-II expression. (**D**) Quantitative analysis of apoptotic cell populations. Data are presented as mean ± SE from three independent biological experiments (*n* = 3). Statistical significance was assessed using one-way ANOVA followed by Tukey’s multiple-comparison test; ** *p* ≤ 0.01; *** *p* < 0.001.

As a result of 48 h exposure to chloroquine (CQ) in HeLa and A549 cell lines, significant changes in the LC3-II protein level were demonstrated, which was determined at 144.05% (*p* ≤ 0.0001) and approx.123% (*p* ≤ 0.0001), respectively (Figure 7(B1,B2),C). However, the level of apoptosis was 41.55% (*p* ≤ 0.0001) for HeLa cells and 30.25% (*p* ≤ 0.0001) for A549 cells (Figure 7(B1,B2),D). In turn, exposure of cells to fexofenadine at a concentration of 300 µM increased both the expression of LC3-II protein to over 160% (*p* ≤ 0.0001) in both tested cell lines and the level of apoptosis to 62.3% (*p* ≤ 0.0001) in cervical cancer cells and to 50.2% (*p* ≤ 0.0001) in lung cancer cells (Figure 7(B1,B2),C,D). The combined action of chloroquine (100 µM) and fexofenadine (300 µM) induced a significant reduction in LC3-II expression to 71.6% (*p* ≤ 0.0001) in HeLa cells and to 90.52% (*p* ≤ 0.0001) in A549 cells, with a simultaneous significant increase in the percentage of apoptotic cells to 91.3% (*p* ≤ 0.0001) and 83.85% (*p* ≤ 0.0001), respectively, with a predominance of the late-apoptotic phenotype (Figure 7(B1,B2),C,D).

The inhibition of autophagy by chloroquine was confirmed by the presence of numerous vacuoles in the cytoplasm of the examined cells, which were clearly intensified as a result of prolonged exposure (Figure 7(A1,A2)). In turn, the combined action of fexofenadine and chloroquine induced an intensification of the apoptosis process, which was expressed by the presence of numerous rounded and shrunken cells, with a simultaneous reduced vacuolization of the cytoplasm (typical of autophagy), and these changes were more intense as a result of 48 h exposure to the tested compounds (Figure 7(A1,A2)).

Collectively, these findings suggest that autophagy serves a predominantly cytoprotective role in fexofenadine-treated cells. Pharmacological inhibition of autophagy by chloroquine markedly enhanced apoptotic cell death, indicating that autophagic responses may partially buffer fexofenadine-induced cellular stress and delay apoptotic progression.

## 4. Discussion

The present study demonstrates that fexofenadine profoundly alters cellular redox homeostasis and survival signaling in cervical and lung cancer cells. The observed biological response was characterized by ROS accumulation, mitochondrial dysfunction, attenuation of PI3K/AKT and MAPK/ERK signaling, activation of apoptotic pathways, and modulation of autophagy-associated processes. Importantly, the ability of N-acetyl-L-cysteine to partially reverse several of these effects supports a functional role for oxidative stress in mediating the cellular response to fexofenadine. Collectively, these findings indicate that disruption of redox homeostasis represents a central upstream event coordinating multiple downstream responses to fexofenadine exposure. Cancer progression and therapeutic resistance are driven by complex and interconnected mechanisms, including altered redox homeostasis, enhanced DNA repair, multidrug resistance, and persistent activation of survival pathways such as PI3K/AKT and MAPK/ERK [8,9,10,32,33,34]. The ability of tumor cells to evade apoptosis remains one of the principal obstacles to effective therapy [8]. Therefore, agents capable of simultaneously targeting mitochondrial integrity, redox balance, and survival signaling networks may offer enhanced therapeutic potential.

Our data demonstrate that fexofenadine reduced cell viability in a concentration-dependent manner and robustly induced apoptotic cell death, as confirmed by Annexin V positivity, caspase-3/7 activation, and accumulation of late-apoptotic phenotypes. These effects were reproduced in three-dimensional spheroid models, indicating that the mechanisms observed in monolayer cultures are maintained in a more physiologically relevant cellular environment.

Ultrastructural analysis revealed pronounced mitochondrial alterations, including swelling and disruption of inner membrane organization. These morphological changes were paralleled by mitochondrial membrane depolarization, Bcl-2 inactivation, and activation of executioner caspases. Given the central role of mitochondria as regulators of apoptotic commitment [35,36], these findings indicate that fexofenadine primarily engages the intrinsic mitochondrial apoptotic pathway. Importantly, Bcl-2 overexpression is strongly associated with chemoresistance [37], and its functional inactivation in our model may lower the threshold for mitochondrial outer membrane permeabilization. Thus, mitochondrial dysfunction appears to represent a critical convergence point integrating redox imbalance and survival signaling attenuation into apoptotic execution.

A similar mitochondrial-targeting mechanism has been described for terfenadine in melanoma [38,39], prostate [19], and breast cancer cells [40]. However, in contrast to older-generation antihistamines associated with cardiotoxicity [41], fexofenadine possesses a favorable safety profile [23,24], which may support further investigation of its biological activity in drug repurposing studies.

In parallel with mitochondrial disruption, we observed widening and swelling of the rough endoplasmic reticulum, consistent with organelle stress. Oxidative stress and endoplasmic reticulum stress are tightly interconnected processes that influence cell fate decisions [42,43,44]. In our study, fexofenadine induced significant ROS accumulation, suggesting that redox imbalance contributes to organelle dysfunction and apoptotic progression. Importantly, pharmacological ROS scavenging with N-acetyl-L-cysteine significantly reduced ROS levels and partially attenuated apoptosis. Although apoptosis was not completely eliminated, the significant decrease in annexin-positive populations following ROS neutralization supports the mechanistic involvement of redox imbalance in mitochondrial destabilization and apoptosis. This incomplete rescue suggests that, in addition to ROS-dependent mechanisms, parallel stress-related pathways may also participate in fexofenadine-induced cytotoxicity.

Taken together, these findings support the concept that oxidative stress represents a major, but not exclusive, driver of the observed proapoptotic effects, functionally linking redox imbalance to mitochondrial dysfunction and apoptotic execution. This finding is consistent with the concept that oxidative stress acts as an upstream amplifier of mitochondrial apoptotic signaling rather than a sole initiator of cell death. To further delineate the signaling mechanisms linking oxidative imbalance to mitochondrial dysfunction and apoptotic execution, we assessed the activation status of the PI3K/AKT and MAPK/ERK pathways, which are central regulators of tumor cell survival, redox adaptation, and resistance to apoptosis. Pathway analysis was performed at a concentration of 200 µM, corresponding to the IC_50_ in HeLa cells and a submaximal cytotoxic concentration in A549 cells.

An important limitation that should be considered when interpreting the present findings is the relatively high concentration range of fexofenadine required to induce anticancer effects in vitro. Reported plasma concentrations achieved following standard therapeutic dosing are substantially lower than the concentrations used in the current study. Nevertheless, the use of supratherapeutic concentrations is common in mechanistic in vitro investigations aimed at identifying cellular targets, stress-response pathways, and potential biological activities of repurposed compounds. Therefore, the present study should primarily be viewed as a mechanistic investigation rather than evidence of direct clinical applicability. Future studies should determine whether similar biological effects can be achieved under pharmacologically relevant exposure conditions, including advanced delivery systems, combination therapies, and in vivo experimental models.

Under these conditions, preceding extensive secondary signaling collapse, fexofenadine significantly reduced p-AKT–positive cell populations and attenuated ERK activation. The PI3K/AKT axis is a key regulator of mitochondrial stability, Bcl-2 activity, and resistance to apoptosis. Its suppression may facilitate mitochondrial outer membrane permeabilization and sensitize cells to oxidative injury. The temporal and functional coincidence of AKT attenuation, Bcl-2 inactivation, mitochondrial depolarization, and caspase activation supports an integrated mechanism linking survival pathway inhibition to intrinsic apoptosis. While the temporal hierarchy of these events cannot be definitively established within the scope of this study, the observed convergence of redox imbalance, survival pathway attenuation, and mitochondrial dysfunction strongly supports a coordinated multi-level mechanism of apoptotic regulation. This suggests that disruption of survival signaling not only facilitates apoptotic execution but may also impair the adaptive capacity of cancer cells to withstand oxidative stress. Moreover, the observed attenuation of PI3K/AKT and MAPK/ERK activity may contribute to the reduced ability of cancer cells to compensate for ROS-induced cellular damage.

Although the present findings demonstrate a clear association between ROS accumulation, attenuation of PI3K/AKT and MAPK/ERK signaling, and apoptotic induction, additional studies employing pathway-specific activators, genetic modulation approaches, and protein-level validation will be required to establish the precise causal relationships among these events.

Autophagy modulation emerged as an additional layer of cell stress adaptation. At lower concentrations, fexofenadine promoted autophagic features, whereas higher concentrations shifted the balance toward apoptosis. This concentration-dependent transition is consistent with the concept that autophagy initially acts as a cytoprotective response but may fail under sustained stress [45,46,47,48,49]. Importantly, pharmacological inhibition of autophagy with chloroquine markedly enhanced apoptosis and reduced LC3-II levels, demonstrating that autophagy partially buffers fexofenadine-induced stress. These findings align with therapeutic strategies aimed at combining autophagy inhibitors with cytotoxic agents to enhance apoptotic efficacy [45,50]. Similar sensitization effects have been reported for other antihistamines, including astemizole and cloperastine, in combination with chemotherapeutic agents [20,51]. Moreover, the combined interpretation of ultrastructural observations, LC3-II modulation, and chloroquine-mediated autophagy inhibition supports the involvement of autophagy-associated processes in the cellular response to fexofenadine, although further studies would be required to fully characterize autophagic flux dynamics.

In addition to apoptotic execution, fexofenadine induced DNA double-strand breaks, as evidenced by increased γH2A.X and ATM activation. DNA damage was accompanied by G0/G1 cell cycle arrest and impaired clonogenic growth, suggesting sustained proliferative suppression. Given the established relationship between oxidative stress and DNA damage [52], ROS accumulation may act upstream of genomic instability, thereby reinforcing apoptotic signaling. Furthermore, the compound significantly inhibited migratory capacity, indicating potential anti-metastatic properties [29]. However, this observation should be interpreted with caution because the concentrations used in the wound-healing assay also induced substantial cytotoxicity and apoptosis. Therefore, the observed reduction in migration may partially reflect decreased cell viability and proliferation in addition to a direct effect on migratory behavior. Future studies employing non-cytotoxic concentrations and complementary migration-specific assays will be required to further clarify the contribution of these mechanisms.

Importantly, evaluation in non-tumorigenic Beas-2B cells revealed markedly lower sensitivity to fexofenadine-induced cytotoxicity and apoptosis. This differential response suggests preferential vulnerability of malignant cells, which are characterized by elevated basal oxidative stress and greater dependence on pro-survival signaling networks [8,9,10,52]. Such selectivity supports the potential translational relevance of our findings and supports the hypothesis that fexofenadine exploits tumor-specific redox and mitochondrial liabilities rather than inducing indiscriminate toxicity. This differential sensitivity is particularly relevant in the context of drug repurposing, as it suggests a potentially favorable therapeutic index.

**Figure 8:**
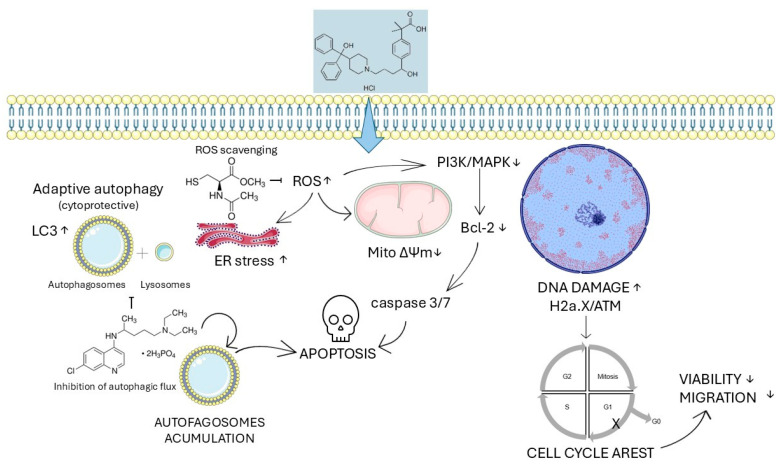
Proposed mechanism of fexofenadine-induced cellular responses in HeLa and A549 cells. Fexofenadine induces intracellular ROS accumulation, leading to mitochondrial dysfunction, attenuation of PI3K/AKT and MAPK/ERK signaling, Bcl-2 inactivation, DNA damage, cell-cycle arrest, and apoptosis. Autophagy-associated responses appear to represent an adaptive cytoprotective mechanism, whereas pharmacological inhibition of autophagy by chloroquine enhances apoptotic cell death. NAC partially attenuates ROS accumulation and apoptosis, supporting the contribution of oxidative stress to the observed biological effects. The symbol “X” indicates blockade of cell cycle progression at the G1/S transition.

Overall, our data support a multi-level mechanism in which oxidative imbalance, attenuation of PI3K/AKT and MAPK/ERK signaling, mitochondrial destabilization, and modulation of autophagy converge to drive intrinsic apoptotic cell death. Collectively, these findings provide mechanistic insight into the biological activity of fexofenadine and support its further evaluation in drug repurposing research. A schematic summary of the proposed mechanism based on the experimental findings obtained in the present study is presented in Figure 8.

Nevertheless, further studies incorporating additional cancer models, protein-level pathway validation, pharmacologically relevant exposure conditions, and in vivo systems will be necessary to determine the translational significance of these observations.

## 5. Limitations

Despite providing detailed mechanistic insights, this study has several limitations. First, the findings are based exclusively on in vitro models and therefore require validation in animal models to determine their biological and translational relevance. Second, only two cancer cell lines and one non-tumorigenic epithelial cell line were investigated. Although these models represent distinct tissue origins, additional cancer and normal cell models would improve the generalizability of the findings.

Third, the concentrations of fexofenadine required to induce anticancer effects in vitro exceed currently reported therapeutic plasma levels. Consequently, the present findings should be interpreted primarily as mechanistic observations, and further pharmacokinetic and in vivo studies are necessary to assess translational feasibility.

Fourth, PI3K/AKT and MAPK/ERK pathway modulation was evaluated using phospho-flow cytometric analysis. Although this approach provides quantitative assessment of pathway activation, additional validation using complementary techniques such as Western blotting would further strengthen the mechanistic conclusions.

Fifth, while NAC rescue experiments demonstrated an important contribution of oxidative stress to the observed cellular responses, additional genetic and pharmacological approaches would be required to fully establish the causal hierarchy between ROS generation, survival pathway modulation, mitochondrial dysfunction, and apoptosis.

Finally, although autophagy-associated responses were evaluated using ultrastructural analysis, LC3-II assessment, and chloroquine-mediated autophagy inhibition, additional studies investigating autophagic flux would provide a more comprehensive understanding of the role of autophagy in fexofenadine-treated cells.

These limitations should be considered when interpreting the present findings and represent important directions for future research.

## 6. Conclusions

In conclusion, fexofenadine induces a coordinated cellular response involving ROS accumulation, mitochondrial dysfunction, DNA damage, and reduced activation of PI3K/AKT and MAPK/ERK signaling pathways. Functional rescue experiments using N-acetyl-L-cysteine identify oxidative stress as a major contributor to the observed biological effects. The demonstrated activity in both monolayer and spheroid cultures, together with the lower sensitivity of non-tumorigenic cells, provides additional insight into the biological actions of this clinically established antihistamine and supports further investigation of its potential in drug repurposing research. Given the in vitro nature of the present study and the concentrations required to achieve the observed effects, further pharmacokinetic and in vivo studies are necessary to determine the translational relevance of these findings.

## Data Availability

The data presented in this study are available from the corresponding authors upon reasonable request.

## Supplementary Materials

The following supporting information can be downloaded at: https://www.mdpi.com/article/10.3390/cancers18132156/s1, Figure S1: Additional flow cytometric analyses omitted from the main manuscript for clarity; Figure S2: Effect of fexofenadine on non-tumorigenic Beas-2B cells.
