# Supplementary material for: Fexofenadine Induces ROS-Dependent Mitochondrial Dysfunction and Suppresses PI3K/AKT and MAPK Signaling in Cervical and Lung Cancer Cells
Source: Cancers (Basel). 2026 Jul 4;18(13):2156. doi: 10.3390/cancers18132156 (PMC13359604; doi:10.3390/cancers18132156)

Supplementary Figure S1. Additional flow cytometric analyses omitted from the main manuscript for clarity.

Representative flow-cytometric histograms corresponding to intermediate concentrations of fexofenadine that were removed from the main figures to improve readability and panel size. Histograms show apoptosis (Annexin V/7-AAD), caspase-3/7 activation, Bcl-2 inactivation, DNA damage, reactive oxygen species generation, and mitochondrial membrane depolarization in HeLa and A549 cells.

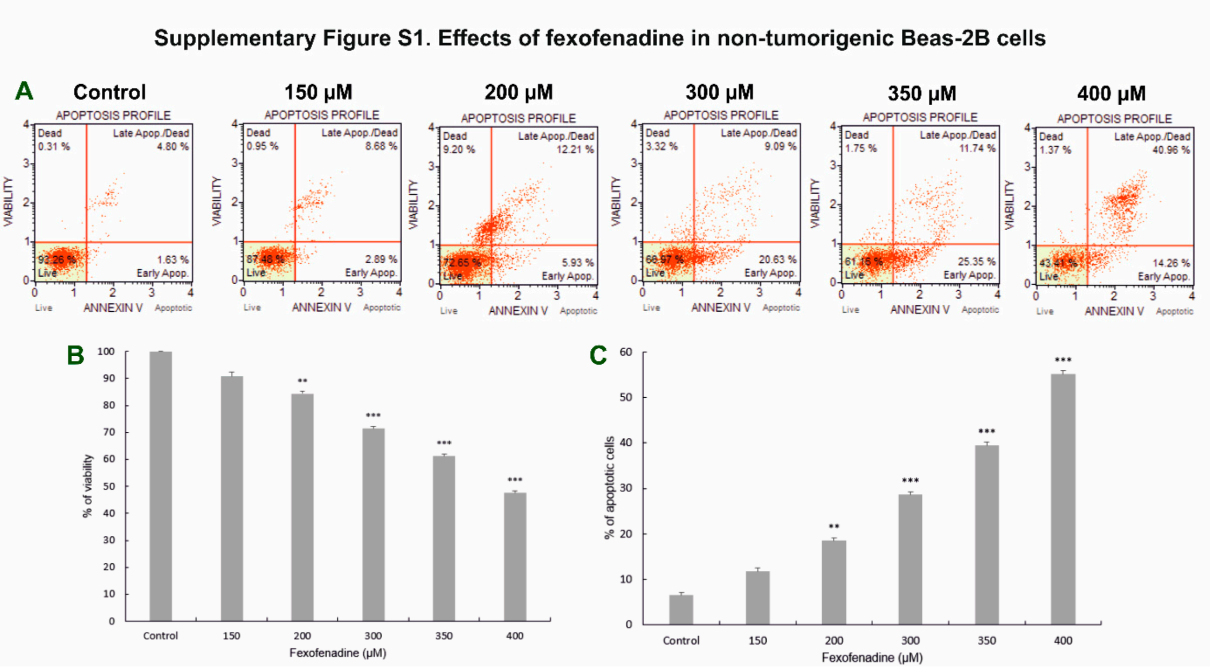

Supplementary Figure S2. Effect of fexofenadine on non-tumorigenic Beas-2B cells.

Representative analyses of cell viability and apoptosis in Beas-2B cells following exposure to fexofenadine. These data demonstrate lower sensitivity of non-tumorigenic epithelial cells compared with HeLa and A549 cancer cells under identical experimental conditions.

Supplementary Figure S2. Additional flow cytometric analyses

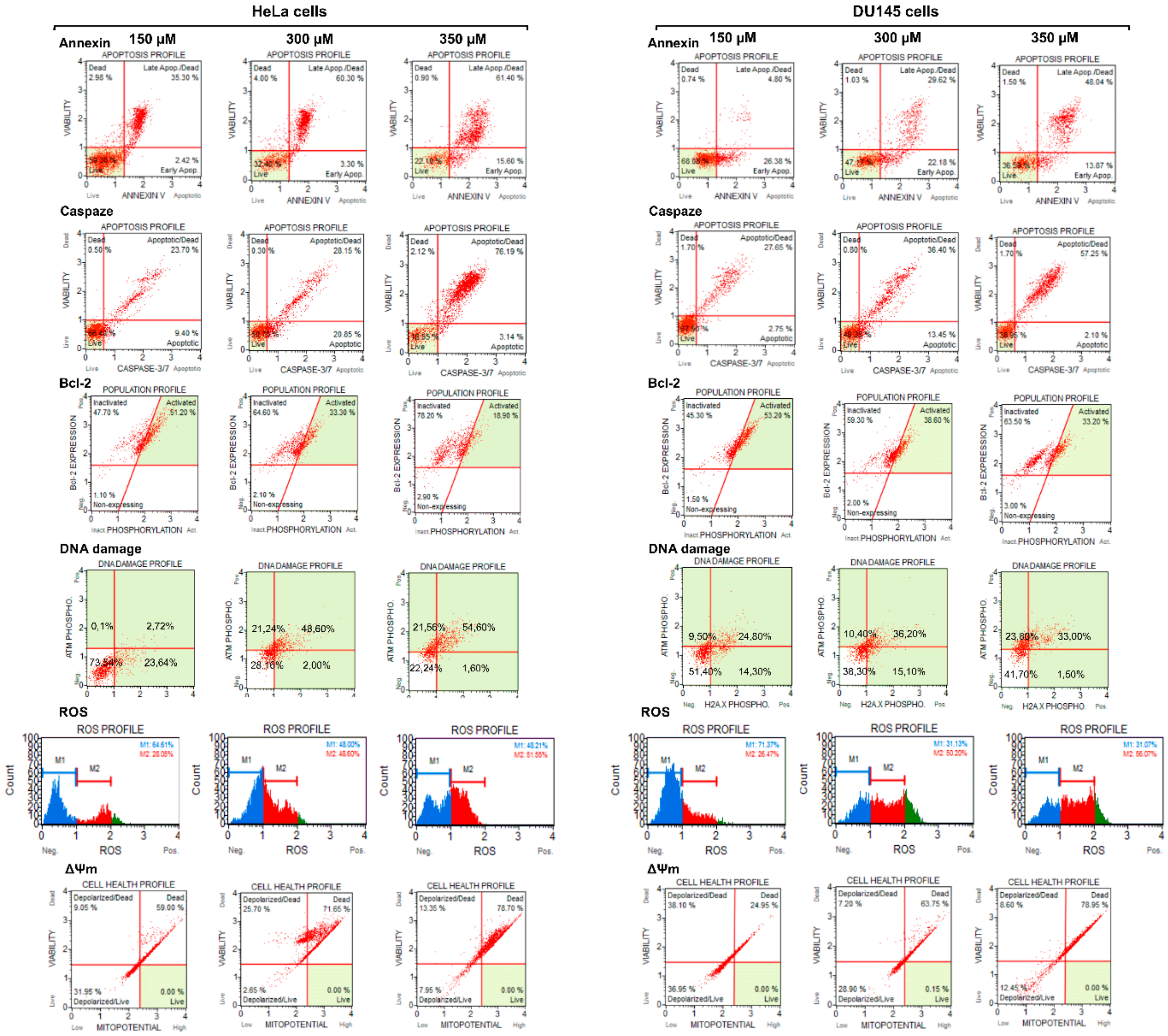

Supplement: Supplementary file 1 [file cancers-18-02156-s001.zip › cancers-4401119-supplementary.pdf]
